# Disruption of cancer cell functions by task-specific drug perturbations

**DOI:** 10.3389/fphar.2022.934843

**Published:** 2022-08-04

**Authors:** Mahmoud Ahmed, Deok Ryong Kim

**Affiliations:** Department of Biochemistry and Convergence Medical Science, Institute of Health Sciences, Gyeongsang National University College of Medicine, Jinju, South Korea

**Keywords:** cancer hallmarks, archetype analysis, gene expression, task specialization, drug perturbations

## Abstract

Cancer expands clonally, capitalizing on the variations between growing cells. Cancer cells specialize in one or more functions to gain an advantage. This study examined the prediction that cells would be vulnerable to drugs that perturb their specific tasks. We analyzed the correlation between gene expression and the response to drug perturbations in different cancer cells. Next, we assigned every cancer cell to an archetype based on gene expression. Finally, we calculated the enrichment of the cancer hallmark gene sets in each cell, archetypes, and response to drug treatment. We found that the extremes of gene expression were susceptible to change in response to perturbations. This correlation predicted the growth rate inhibition of breast cancer cells. Cancer hallmarks were enriched differently in the archetypes, and this enrichment predicted the cell’s response to perturbations. We present evidence that specialized cancer cells are sensitive to compounds that perturb their tasks.

## 1 Introduction

Tumor cells share a core functionality that separates them from normal cells (Hanahan and Weinberg, 2011). Cancer expands clonally, benefiting from the variations between the growing cells (Nowell, 1976; Greaves and Maley, 2012). Clones consist of identical cells; these differ from the surrounding cells. These similarities and differences exist at the level of genomic content and gene expression. Inevitably, the variations drive differential allocation of resources toward some functions. A recent study has shown that in their distinct life histories, cancer cells make trade-offs between cellular tasks or functions (Hausser et al., 2019). As a result, cells specialize in one or more tasks to their advantage. One prediction of this settlement is that cancer cells would be susceptible to drugs that perturb these specialized tasks. The authors observed that the distance between cancer cells and a typical gene expression profile characteristic of a given task predicted their growth rate when treated with a drug that disrupts the same task.

Cancer cell lines represent variations of tumors derived from the same tissue (Gillet et al., 2013). They arise in different cell types, give rise to various diseases, and exhibit diverse behavior. Thus, the cell lines potentially have distinct life histories characterized by trade-offs similar to their tumor of origin. However, it is an open question whether specializing and task switching events can be detected in the cell expression profiles. This would unlock the possibility of observing the specialization and trade-offs at a granular level. Consequently, functions can easily be defined as a gene set or a collection of proteins performing a particular task or forming a pathway.

Several large-scale datasets were generated to profile genomic and transcriptomic responses to cancer drugs (Barretina et al., 2012; Garnett et al., 2012; Seashore-Ludlow et al., 2015). Different approaches were applied to predict and explain the efficacy of these drugs in particular types of cancer. These approaches ranged from identifying critical changes associated with drug responses to using deep neural networks (Iorio et al., 2016; Chang et al., 2018). A unifying theory of why particular cancers are susceptible or sensitive to certain drugs is still lacking. In addition, the increase in chemo-resistance is almost inevitable in almost all types of cancer treatments (Easwaran et al., 2014). Therefore, further research should be directed at not only predicting effective drugs but also understanding the principles behind cancer cell susceptibility.

Here, we investigate drug sensitivity to specialized disruption using expression profiles of cancer cell lines. We analyzed the correlation between gene expression and changes in response to drug perturbations. We performed an archetype analysis of the typical gene expression profiles of cancer cells to find the extremal that represents pure expression patterns. We assigned every cancer cell to an archetype based on the shortest distance. Finally, we calculated the enrichment of the genes involved in the cancer hallmarks in each archetype and cell response to drugs.

## 2 Materials and methods

### 2.1 Gene expression data of drug perturbations in cancer cell lines

The library of integrated network-based cellular signatures (LINCS) is a collection of gene expression profiles of cancer cell lines under different types of perturbations, including compounds, gene overexpression, knockdown, or knockouts (Koleti et al., 2018). Cancer cell lines (*N* = 46) were treated with drugs (*N* = 38; for which drug sensitivity data are available) or DMSO (controls) and profiled for gene expression using the L1000 technology, which only measures the expression of 1,000 genes and imputes the expression of the remaining from these measurements. The data were obtained using the slinky R package (Kort, 2021).

### 2.2 Growth inhibition of MCF7 with drug perturbations

We obtained cell viability measurements under treatments with different drugs (*N* = 39; for which gene expression data are available) in breast cancer cell lines (Hafner et al., 2016). The data are presented in the form of the maximum growth inhibition GRmax values of each drug which takes into account the baseline replication rate. These values were plotted against the correlations between the average gene expression profile of the cell lines and their characteristic direction (CD) of change in response to treatments with the same drugs (details in subsection 2.6).

### 2.3 Cancer hallmark gene sets

Lists of genes involved in the ten cancer hallmarks were previously compiled (Zhang et al., 2020). The lists were manually curated from 301 KEGGs pathways (Kanehisa et al., 2017). The creators of the list related each of the pathways to one of the ten cancer hallmarks using text mining. After manual confirmation, the lists were filtered and tested against mutation, methylation, and copy number variation data from cancer tissues. We used the lists of hallmarks as gene sets and calculated the enrichment of each in the archetypes and the cell line responses to drugs (details in subsection 2.7).

### 2.4 Multidimensional scaling (MDS)

Control samples of the cell lines were used to determine their typical gene expression profiles by averaging across replicates. Multidimensional scaling (MDS) was applied to show the similarity between the gene expression profiles of the cell lines (Cox and Cox, 2000). The pairwise distances between the averaged gene expression profiles of the cells were calculated and projected on a two-dimensional space. Individual points were labeled using different variables (tissue of origin or archetypes) to decide which explained more variance among the expression profiles.

### 2.5 Archetype analysis (AA)

Archetype analysis represents a set of observations as convex combinations of pure patterns. Individual observation will either belong to one of the pure archetypes or be a mixture of two or more. A set of archetypes was learned from the typical expression profiles so that the data are approximated by a convex combination of the extremal points (Cutler and Breiman, 1994). The problem is defined and solved as context least squares using linear equations. We recalculated the model for a different number of archetypes and chose the one (*k* = 6) with a small residual sum of squared errors. The nearest archetypes were designated based on the sum of the square differences between the model parameters (genes) and various archetypes. The model parameters were entered into the enrichment analysis as ranking values of the features (genes) in the archetypes. This analysis was applied using the archetypes R package (Eugster and Leisch, 2011).

### 2.6 Differential expression analysis (DE)

The CD of change between treated and control (DMSO) samples was compared to obtain the differential gene expression. This analysis was applied using the diffexp function from the slinky R package (Kort, 2021). This function uses the characteristic direction (CD) method which gives less weight to individual genes that exhibit a large change in the magnitude between two conditions. Instead, the method gives more weight to genes that move together in the same direction across repeats (Clark et al., 2014). The sign of the CD represents the direction of change and the magnitude of the importance of the corresponding gene in the cell response to drug perturbations (Duan et al., 2016).

### 2.7 Gene set enrichment analysis (GSEA)

The parameters of the six archetype models were used to rank the genes. The lists of genes were also ranked by the magnitude of CD in response to drug treatments in each cell. Enrichment scores were calculated in each list with the over-representation of the cancer hallmark gene sets at the top or the bottom of the list. This approach allows the detection of small changes in gene expression in multiple members of the gene set. Enrichment scores were considered significant when the false-discovery rate (FDR) was < 0.2. This analysis was applied using the clusterProfiler R package (Yu et al., 2012).

### 2.8 Correlation analysis

**FIGURE 1:**
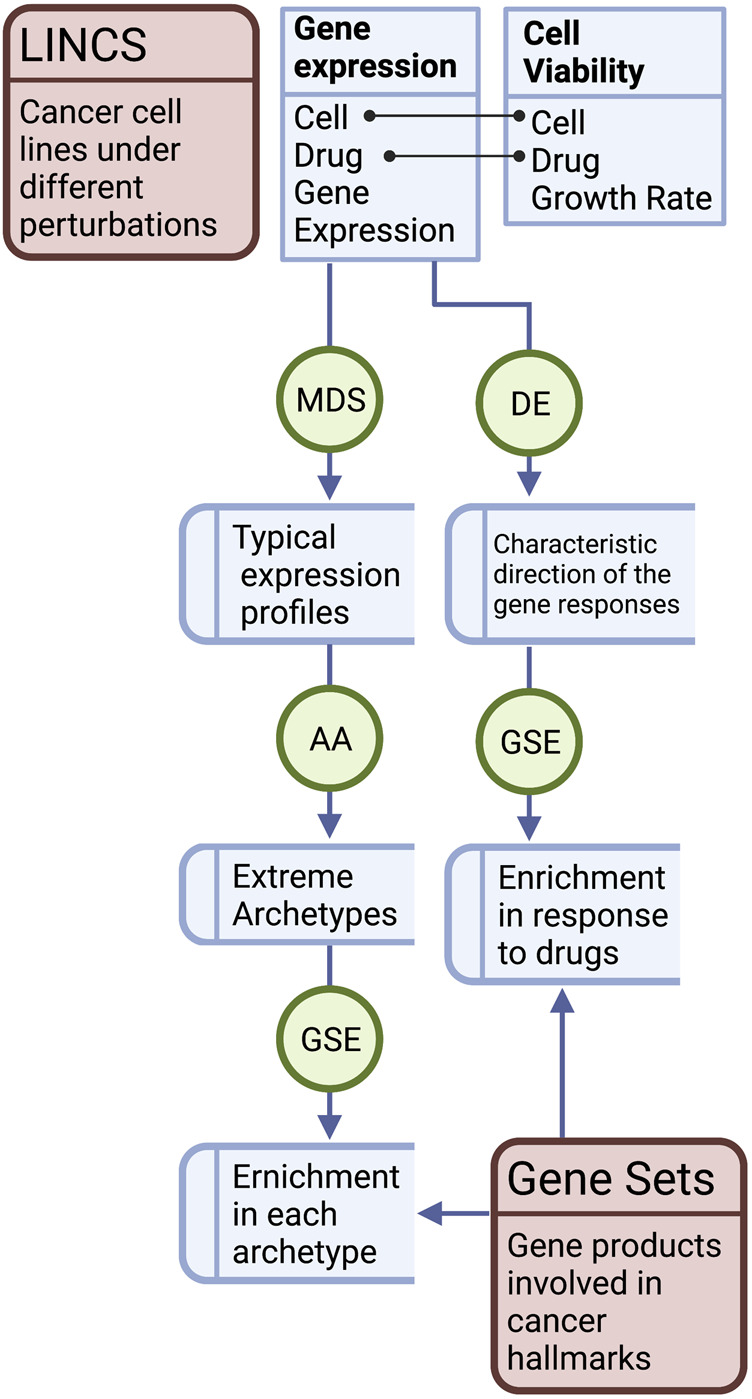
Workflow of the study. Gene expression data of the cancer cell lines were used to determine the typical expression profiles and the response to treatments in the form of differential expression (DE) characteristic direction (CD) of change. Typical gene expression profiles of the cancer cell lines were subjected to multidimensional scaling (MDS), archetype analysis (AA), and correlation analysis with the CD of the drug treatments. The correlations in MCF7 were plotted against growth inhibition rates of the cell line treated with the same set of drugs. Gene set enrichment analysis (GSEA) of the cancer hallmarks was performed in the pure archetypes of the cell lines and their responses to drugs was analyzed. The correlation between the enrichment scores of the archetypes and the drug response was analyzed.

The variance explained by different variables in the gene expression profiles was estimated as intra-class correlation (ICC) using the variancePartition R package (Hoffman and Schadt, 2016). The correlation between the typical expression profiles and the response to drug perturbation (CD) was calculated using Spearman’s rank coefficients. The correlation (discretized) of expression and responses in MCF7 were plotted against the growth inhibition when applying the same treatment. The correlation between the enrichment scores of the cancer hallmark gene sets in the archetypes and the drug responses was calculated using the Pearson correlation coefficient (PCC). These correlations were plotted against the absolute enrichment scores (discretized) of the archetypes. Figure 1 shows a diagram of the workflow of the study.

### 2.9 Data, source code, and reproducibility

The data analyzed in this study were obtained from online repositories: https://www.ncbi.nlm.nih.gov/geo/query/acc.cgi?acc=GSE92742 and https://lincs.hms.harvard.edu/db/datasets/20268. The software environment and code to reproduce this analysis is available as a docker image (https://hub.docker.com/r/bcmslab/task_perturbation) and an open-source compendium (https://github.com/BCMSLab/task_perturbation). The analysis was conducted in R (4.0) using Bioconductor (3.11) (R Core Team, 2021; Huber et al., 2015).

## 3 Results

### 3.1 Gene expression extremes are susceptible to drug perturbations

We evaluated the relationship between the typical expression profiles of the cancer cells and their response to drug perturbations. We defined a “typical” expression profile as the average gene expression in the control cells (treated with DMSO) (*N* = 14) and the “response” as the characteristic direction (CD) of change between cells treated with each drug (*N* = 38) and the control. Each of the two variables was represented as a vector with a length equal to the number of genes and the association measured between them. To calculate the per cell line or drug correlation, we averaged the response of the cell line to all drugs or the response to the drug across the cell lines.

**FIGURE 2:**
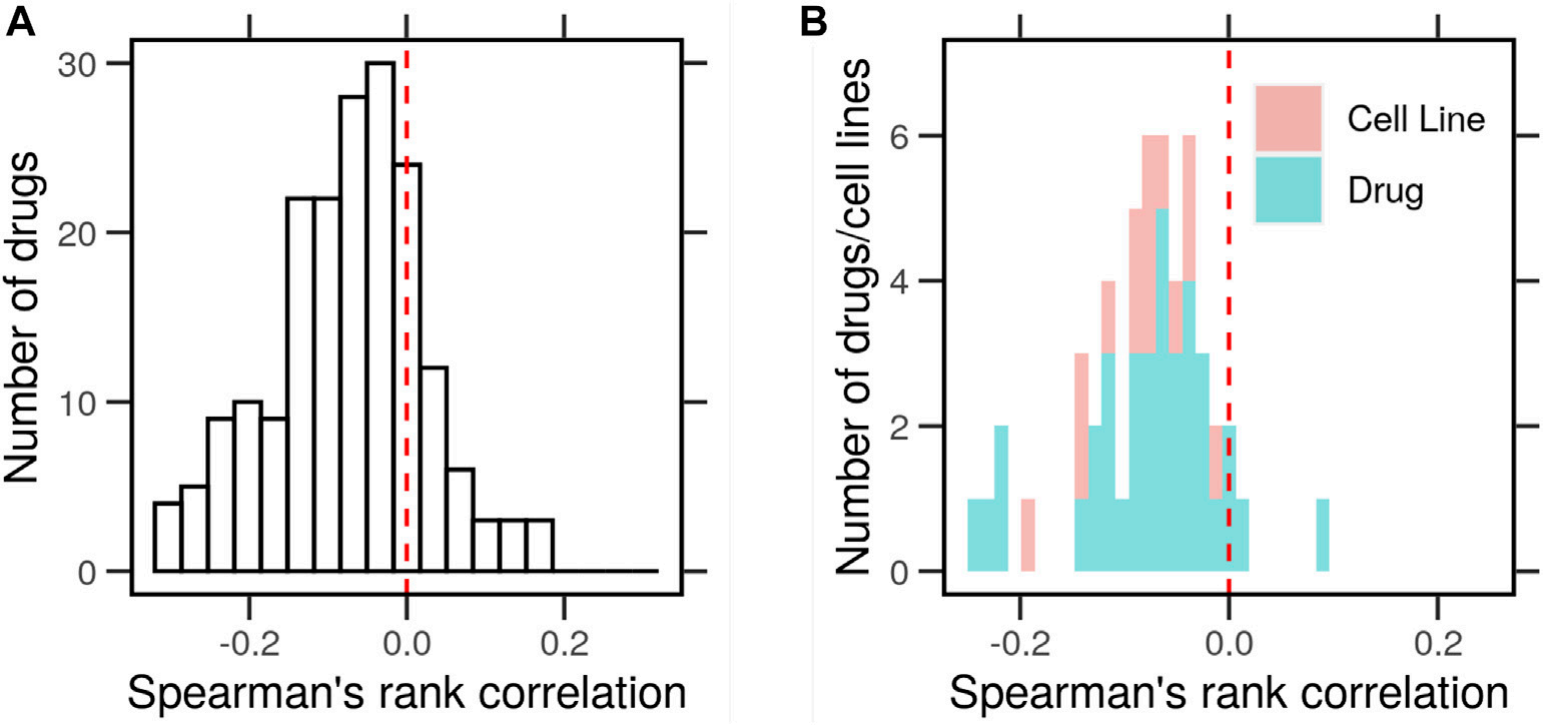
Correlation between the typical gene expression of cancer cells and the change in response to drug perturbations. Gene expression profiles of cancer cell lines (*N* = 46) with and without drug perturbations (*N* = 38) were obtained from the library of integrated network-based cellular signatures (LINCS). The typical expression profiles were determined as the average by the cell line in the control conditions (DMSO). The characteristic direction (CD) of change between treated and control cells was determined. **(A)** Histogram of Spearman’s rank correlation between the average gene expression and the CD. **(B)** Histogram of the correlations averaged for every drug (blue) or cell line (red).

The correlation (Spearman’s rank correlation coefficients) between the typical expression profile and drug response in terms of gene expression was moderately negative but significant (*ρ* < 0; *p* < 0.05) for the majority of the data points (70%). A histogram of the correlations shows a shift to the left, indicating that genes at the extremes of the expression distributions tend to change in response to drug treatments (Figure 2A). We compared the distribution of the correlations to that of a normally distributed random variable centered around zero using the Kolmogorov–Smirnov (KS) test. The cumulative distribution of the two curves was significantly apart (*D* ^−^ = 2.14, *p* < 0.0001), indicating that they were drawn from the distribution of different shapes.

Next, we asked whether the negative correlation between the typical gene expression and drug responses holds for every cell line and drug. We separated the histogram of correlations by the cell line or drug. In both cases, the correlation in question was negative (*ρ* < 0; *p* < 0.05) in most cell lines (95%) and for most drugs (90%) (Figure 2B). In both cases, the cumulative distributions of the average correlations were significantly different (*D* ^−^ = 1.4 *&* 1.8, *p* < 0.0001) than those from a random variable in the KS test. This shows that the extremely highly and lowly expressed genes in each cell changed the most in response to drug treatment in terms of their expression. Next, we clustered these expression profiles to look for patterns that could explain their correlation with the drug perturbation response.

### 3.2 Archetypes explain the variance in expression among cancer cells

**FIGURE 3:**
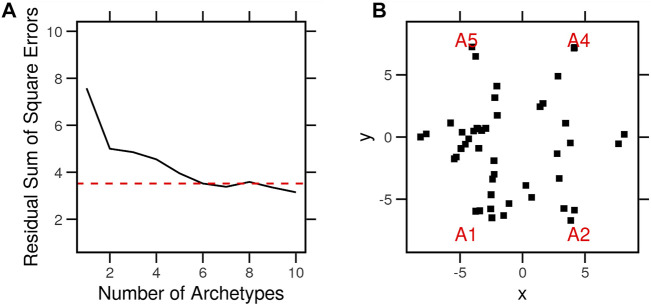
Archetypes of the typical gene expression profiles of cancer cell lines. Typical expression profiles were determined by averaging the control condition of each cell line (*N* = 46) in the LINCS dataset. A set of archetypes was determined from the typical expression profiles so that the data are approximated by a convex combination of the extremal points. The problem is defined and solved as context least squares using linear equations. **(A)** Procedure was repeated with several archetype numbers, and the residual sums of square errors were calculated. **(B)** Archetypes (red) and cell lines (black) in parallel coordinates of a model with six archetypes.

We used archetype analysis to find the extremes of expression profiles across non-treated cancer cell lines (*N* = 46). These profiles were determined so that the average expression profiles of the cell types are approximated by a convex combination of extremal points (archetypes). The archetypes represent the pure expression profiles to which individual cells belong (specialists) or are a mixture of two or more (generalists). Clustering, by contrast, identifies typical profiles (centroids) to which other observations are assigned based on some measure of distance. We selected six (*K* = 6) archetypes to minimize the residual sum of squares (*RSS* = 3.5) and retained the possibility of interpretation (Figure 3A). With the number of archetypes, many of the cancer cells were on or nearby one archetype (Figure 3B). Most cells, however, were on the line between two of these six archetypes. This is to say, the cell does not belong strictly to either but comprises features of each (Table 1). For example, HS578 T belonged to the fifth archetype (A5), while MCF7 was a mixture of archetypes (A1, 2, and 6). The archetypes A1 and A6 were common to most cells.

**TABLE 1:** Cancer cell line membership in different archetypes.

	Cell line	A1	A2	A3	A4	A5	A6
Breast	BT20	X					X
	HS578 T					X	
	MCFX0A	X	X			X	X
	MCF7	X	X				X
	MDAMB23X	X				X	X
	SKBR3	X					X
Blood/lymphoid	HL60				X		
	NOMOX		X		X		X
	PHH		X				
	PL2X	X			X		
	SKMX	X			X		
	THPX				X		X
	U937				X		
	WSUDLCL2	X			X		
Large intestine	CL34		X				X
	HCTXX6	X	X				X
	HTXX5						X
	HT29	X	X				X
	LOVO	X	X				X
	MDST8	X					
	NCIH508	X				X	
	RKO			X			
	SNUX040				X		
	SNUC4	X	X				X
	SNUC5		X				X
	SW480	X				X	X
	SW620	X					X
	SW948		X				X
Lung	A549	X	X			X	X
	CORL23		X				X
	DV90	X		X			X
	HX299			X			
	HCCX5	X				X	X
	HCC5X5	X				X	X
	NCIHX694						
	NCIHX836		X				
	NCIH2073	X					X
	NCIH596	X					X
	SKLUX					X	
	T3MX0	X	X				X
Ovary	COV644		X				
	EFO27	X					X
	OV7	X	X				
	RMGI	X					X
	RMUGS	X	X				X
	TYKNU	X	X				X

We calculated the differences between the gene expression values (feature) in each cell and the archetype model parameters. The cells were assigned to one or more archetypes based on the sum of squared differences (*SSD* < 120) (Table 1), which represent the distance of the cell from the pure archetypes. For the purpose of the following analyses, we assigned each cell to the closest archetype (the lowest SSD). If these archetypes reflected anything important about the different cells, it would have been expressed as better clustering or explained a larger amount of variance between the cells than that of other known variables (such as tissue of origin).

**FIGURE 4:**
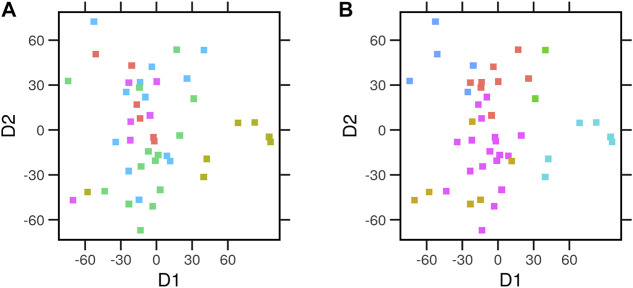
Multidimensional scaling of the average expression profiles of cancer cell lines. Gene expression for the control condition of the library of integrated network-based cellular signatures (LINCS) cell lines (*N* = 46) data was used in multidimensional scaling (MDS). Euclidean distances between the expression profiles were calculated. The relative position of the cell lines across the first two dimensions (D1 and D2) is laid out. Each point represents a cell type. Cells were colored by the different **(A)** tissue of origin or **(B)** the assigned archetype.

We performed multidimensional scaling (MDS) on the average expression profiles of every cell. We then assigned each a tissue of origin (Figure 4A) and an archetype (Figure 4B). Cells clustered better across the two dimensions when labeled by the nearest archetypes. To quantify this observation, we considered the so-called intra-class correlation (ICC) as a measure of the fraction of variance explained by the tissue of origin or archetype. Archetypes explained a higher proportion (*ICC* = 0.28) of the variance between the gene expression profiles of the cells than the tissue of origin (*ICC* = 0.06). Next, we explored whether the archetypes differ from each other in terms of the enrichment of the cancer hallmark gene sets.

### 3.3 Cancer hallmark enrichment differs between cell archetypes

**FIGURE 5:**
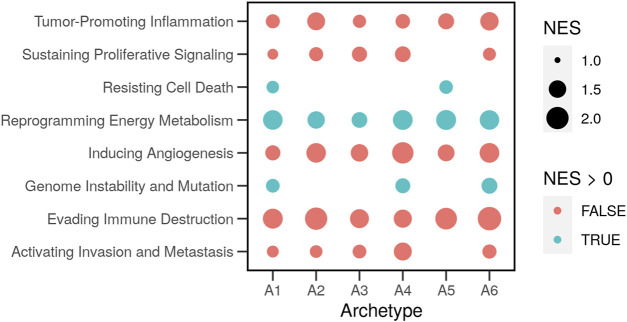
Enrichment of the hallmark gene sets in the archetypes of cancer cells. Genes known to be involved in the ten cancer hallmarks were compiled in gene sets (Zhang et al., 2020). Gene expression profiles of cancer cell lines (*N* = 46) with and without drug perturbations were obtained from the library of integrated network-based cellular signatures (LINCS). The characteristic direction (CD) of change between treated and control cells was determined. Genes were ranked based on the CD, and the overrepresentation of the hallmark gene sets (normalized enrichment scores; NES) was estimated in the top (*NES* > 0) or the bottom (*NES* < 0) of the ranked list.

The hallmark gene sets were previously compiled from lists of genes involved in the relevant functions and pathways. The aforementioned analysis assigns each feature (gene) a value that indicates its contribution to each archetype. We used the model parameters of the features to rank genes and calculate the gene set enrichment scores of the cancer hallmarks in each archetype (Figure 5). We found evidence of significant differences with a small false-discovery rate (*FDR* < 0.2) for eight out of ten hallmarks. For example, the set of genes involved in “Reprogramming Energy Metabolism” was overrepresented in the list of highly expressed genes in all cell archetypes. This was evident in a significant (*FDR* < 0.001) positively normalized enrichment score (*NES* = 1.4–1.7). In other words, the members of the gene set were not randomly distributed in the ranked list.

The two gene sets: “Resisting Cell Death” and “Genome Instability and Mutations” were only enriched (*NES* = 1.2 *&* 1.3; *FDR* < 0.08 *&* 0.6) in two or three archetypes. Other gene sets were overrepresented lower in the ranked list of expressed genes. These observations show the relative contributions of genes to each archetype. The difference itself is relative between the archetypes and the genes since genes not expressed were removed in advance. The process of clustering into archetypes was then used to find the differential response to drugs in the functions that are important for each archetype.

### 3.4 Cancer cells are sensitive to drug perturbations that disrupt their specialized tasks

**FIGURE 6:**
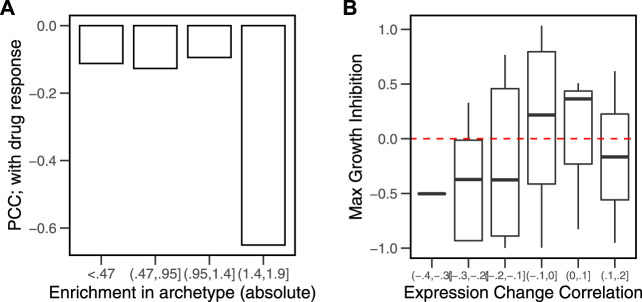
Correlation between hallmark enrichment in archetypes and in response to drug perturbations. A set of six archetypes was estimated from gene expression data of cancer cell lines (*N* = 46) obtained from the library of integrated network-based cellular signatures (LINCS). The model parameters (alphas) were used to rank the genes in a list. The characteristic direction (CD) of change between the control and drug perturbed (*N* = 38) cell lines were used to obtain a ranked list of genes. The overrepresentation of the hallmark genes sets at the extremes of the archetype parameters and the drug response lists was calculated. **(A)** Pearson’s correlation coefficients (PCCs) between the two enrichment scores for each group of absolute archetype enrichment values. **(B)** Correlations between the average gene expression profile in MCF7 and the CD in response to drug perturbations were calculated. Growth inhibition data for the same set of drugs were obtained by Hafner et al. (2016). Growth inhibition for drugs across the range of correlations is shown as boxplots.

The previous analysis identified the cancer hallmarks (tasks) that are important in each archetype (enrichment in archetypes). Using CD to rank the genes, we calculated similar enrichment scores for the gene sets as the different cells responded to perturbations (enrichment in response to drugs). We then calculated the Pearson correlation coefficients (PCCs) between the enrichment scores for each cell in the hallmarks related to its nearest archetype. We found that correlation is the strongest (*r* = −0.6, *p* < 0.01) when the enrichment of the hallmark in the archetype is the highest (*NES* > 1.4) (Figure 6A). The more pronounced the enrichment of a biological function in the cell is, the bigger the expression change of the genes involved in it will be.

A similar observation could be derived for the susceptibility of individual genes to change in response to perturbations. The average gene expression in cancer cells and their response to drug perturbations were associated with growth inhibition as a result of treatment with the same drugs (Figure 6B). The most negative correlations (*ρ* < − 0.1, *p* < 0.05) occurred in drug treatments that produced higher growth inhibition (*GRmax* < 0). Treatment with a given drug was more effective in inhibiting cell growth when the extremes of gene expression were susceptible to the drug, that is, the drugs that produce the largest effect on the highest and lowest expressed gene inhibit cell growth the most.

## 4 Discussion

**FIGURE 7:**
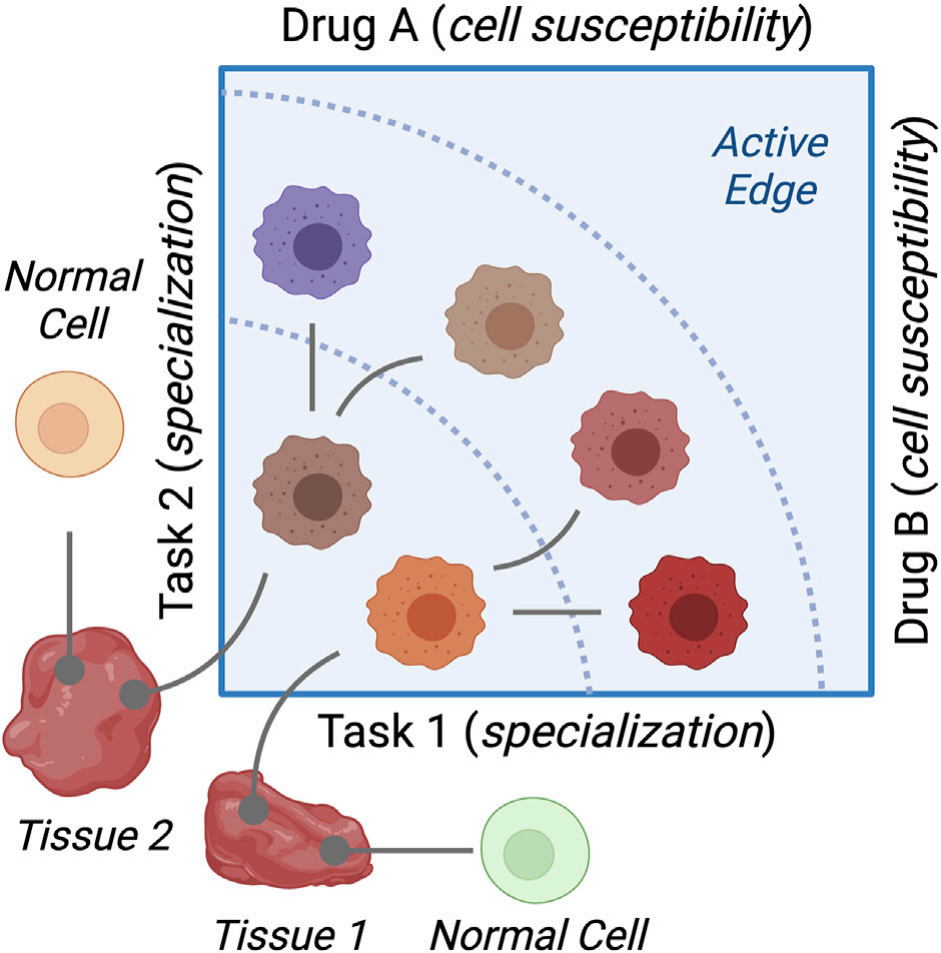
Diagram of the study findings. Cancer cell lines represent variations of cancer cells growing in different tissues. Resources dedicated to specific cellular functions differ between the cell types. This results in cancer cells specializing in functions (tasks) and making trade-offs to their advantage. Cells specialized in a given task are sensitive to drugs that disrupt this task.

This article shows that cancer cells that are specialized for a particular task are susceptible to drugs that interfere with that task. First, the extremes of gene expression were more susceptible to change by drug treatments. Dependent on these changes was the growth rates of breast cancer cells under the same treatments. Second, cancer cells are clustered in archetypes that represent extreme gene expression profiles. Cancer hallmarks were enriched discrepantly in these archetypes, and this enrichment was negatively correlated with the enrichment in response to drug perturbations. Figure 7 summarizes the findings and conclusions of the study.

Cancer cells compete for resources among each other and with normal cells in the body. Therefore, a limited supply of resources and metabolic constraints suggest trade-offs that might exert selective pressures. Experiments in breast cancer cells have shown a reduction in proliferation rates when the need to detoxify oxygen species arises (Jerby et al., 2012). Similar trade-offs between the metabolism of serine and glutamine have been observed in estrogen-positive versus estrogen-negative cells. Aktipis and others provided a perspective on applying life-history theory to the evolution of cancer (Aktipis et al., 2013). In particular, they posited that different hallmarks of cancer are associated with faster or slower life histories depending on the stability and availability of resources in the microenvironment.

Simulations were used to explain how such task trade-offs could work in the context of cancer. Driven by a hypoxic microenvironment, glioblastoma cells switch from proliferative to invasive phenotype (Hatzikirou et al., 2012), rather than being transformed to the more aggressive form by mutations. Godlewski et al. suggested a possible mechanism for phenotype switching in glioma cells (Godlewski et al., 2010). More recent research studies have made apparent the non-static dynamic nature of tumor growth (Gallaher et al., 2019). In simulated models, the turnover rate in glioma cells affects the tumor growth rate and the proliferation/migration trade-off. Increasing cell turnover slows the overall growth and accelerates the evolution of proliferation in the interior and migration at the edge of the tumor.

The concept of the Pareto front has been used to infer the trade-off between the phenotypes of the organisms that multi-task (Shoval et al., 2012). The authors of this previous study proposed to use the weighted average of the archetypes that specialize in one or more tasks to find the trade-offs. This scheme was applied to high-dimensional gene expression data to infer the biological tasks (Hart et al., 2015). Hausser et al. used the aforementioned approach to study task specialization and trade-offs in tumors (Hausser et al., 2019). Their analyses predict and test how tumors that specialize in a given task are sensitive to drugs that disrupt that task. Several issues were raised about this approach and about the use of static (snapshot) high-throughput data to study the life history of evolving cancer.

Considering many tasks may hinder defining trade-offs for several of the tumors that were included in the study (Hausser et al., 2019). Plutynski pointed out The Cancer Genome Atlas (TCGA) data, on which the study was based, might be biased toward the task that the study explains (Plutynski, 2021). In addition, an analysis of life history and trade-offs should draw on phenotypes as well as genotypes. Our present analysis supports the explicit prediction of the study regarding the drug sensitivity of specialized tumor cells. Gene expression data of cancer cell lines reflect an unbiased and wide range of tumor variations with potentially distinct life histories. We also show that the extremes of gene expression are sensitive to drug perturbations and that this association explained the phenotypic change in the form of reduced growth rates of breast cancer cell lines.

Genes at the extremes of the expression profiles (very high or very low expression) disproportionately contribute to enrichment of the gene sets of which they are part of and to the variability between cell types. In either case, these genes are likely to change in response to perturbations. Our analysis suggests that the susceptibility of specialized cells leads to changes in the enrichment of the gene sets (tasks) and expression of the individual genes. In addition, previous studies have shown that variable genes are more likely to be differentially expressed between conditions. A meta-analysis of gene expression variability in yeast concluded that environmental perturbations, in particular, led to more significant gene expression variability and overall regulation than genetic manipulations (Pancaldi et al., 2010). Moreover, expression variation partially predicted differential expression in response to perturbations in both Drosophila and humans (Sigalova et al., 2020). Finally, in the aggressive subtype of chronic lymphocytic leukemia, high-variability genes were related to cell cycle, development, and intercellular communication gene sets, indicating a relation to faster progression of the subtype (Ecker et al., 2015).

Cell line data, however, are limited by the fact that they represent discontinuous lines of evolution. These can only be studied, at least in the current investigation, as the endpoint or the end product of the process that generated them. In addition, the way we define tasks as hallmarks of cancer is also a potential source of bias. On the other hand, we present a flexible way to define and test for quantitative differences in the tasks in terms of enrichment of gene sets. We use this approach to examine how different molecular functions contribute to the archetype that the cell type belongs to and relate to the drug response. The interpretation of the drug sensitivity in multi-tasking cells depends on the existence of the notion of specialization, which we do not test explicitly in this study.

## Data Availability

Publicly available datasets were analyzed in this study. These data can be found here: https://www.ncbi.nlm.nih.gov/geo/query/acc.cgi?acc = GSE92742.
